# Predictive value of urinary [TIMP-2]•[IGFBP7] for AKI among sepsis, stroke, and cardiac surgery cohorts: A prospective study

**DOI:** 10.1371/journal.pone.0332272

**Published:** 2025-10-10

**Authors:** Chaoyun Jiang, Cheng Yang, Hui Chen, Xiaofang Jiang, Jiahao Zhang, Juan Felipe Alvarez, Haichuan Yu, Yao Zhu, Lianjiu Su, Zhiyong Peng

**Affiliations:** 1 Department of Critical Care Medicine, Zhongnan Hospital of Wuhan University, Wuhan, Hubei Province, China; 2 Department of Critical Care Medicine, Shenzhen Second People’s Hospital, Shenzhen, Guangdong Province, China; 3 Division of Cardiology, Department of Medicine, David Geffen School of Medicine, University of California, Los Angeles, California, United States of America; Guilan University of Medical Sciences, IRAN, ISLAMIC REPUBLIC OF

## Abstract

**Background:** TIMP-2 and IGFBP7 have shown effectiveness as biomarkers for predicting Acute Kidney Injury (AKI). However, the variations in the predictive capacity of urinary [TIMP-2]• [IGFBP7] for AKI across different etiologies remain unexplored. This study aimed to assess the predictive capability of urinary [TIMP-2]• [IGFBP7] for AKI in three distinct disease cohorts (stroke, sepsis, and cardiac surgery) characterized by differing AKI etiologies.

**Methods:** This prospective observational study evaluated the predictive value of urinary [TIMP-2]• [IGFBP7] among three cohorts with varying AKI causes. Binary logistic regression was employed to identify AKI’s independent risk factors and develop a combined prediction model. The predictive value was assessed using Receiver Operating Characteristic (ROC) curves and Area Under the Curve (AUC) analyses.

**Results:** 337 patients were included in the final analysis, with 109 (32.3%) developing AKI. AKI occurred in 39 (22.2%) stroke patients, 52 (50%) sepsis patients, and 18 (31.6%) post-cardiac surgery patients. [TIMP-2]• [IGFBP7] exhibited predictive value for AKI with an AUC of 0.86 (95% CI 0.75-0.90) in stroke, 0.82 (95% CI 0.74-0.91) in sepsis, and 0.90 (95% CI 0.82-0.98) in post-cardiac surgery. DeLong’s test indicated no significant differences in the predictive value of [TIMP-2]• [IGFBP7] between the cardiac surgery group and the stroke (P=0.20) and sepsis (P=0.21) groups.

**Conclusion:** The combined prediction model, which integrates urinary [TIMP-2]• [IGFBP7] concentrations and AKI risk factors, significantly enhances AKI prediction. No significant differences were found in the predictive value of urinary [TIMP-2]• [IGFBP7] for AKI among the stroke, sepsis, and cardiac surgery cohorts.

## Introduction

AKI is defined as an acute decline in kidney function occurring within 7 days, characterized by an increase in serum creatinine or a decrease in urine output [1]. The prevalence of AKI among hospitalized patients ranges from 7.2% to 9.6% [2]. Yet, a multi-center investigation involving nearly 90,000 critically ill individuals revealed that AKI was present in one-sixth of all ICU admissions, significantly affecting patient outcomes[3]. Notable risk factors for AKI in ICU patients include stroke, sepsis, and cardiac surgery, which contribute to both individual and societal healthcare burdens [1,4–6].

The Acute Dialysis Quality Initiative (ADQI) published recommendations in 2020 advocating for including injury biomarkers in the AKI definition [7]. [TIMP-2]• [IGFBP7] are cell-cycle arrest biomarkers released by renal tubular cells during early stress. Their product reflects G1 cell cycle arrest, a protective mechanism against nephron injury, making them sensitive early AKI indicators [8,9]. Unlike injury markers (NGAL/KIM-1), [TIMP-2]• [IGFBP7] reflect cellular stress preceding damage, explaining their earlier rise (2–6h vs. 12–24h) [8,10]. This may enable preemptive interventions. Numerous comparative studies have indicated that the combination of [TIMP-2]• [IGFBP7] offers distinct advantages over other emerging biomarkers in predicting AKI in patients experiencing sepsis, shock, major surgical procedures, and trauma [8,9]. This biomarker combination demonstrates early predictive capabilities in various scenarios, including post-cardiac surgery AKI, septic AKI, and hepatic decompensated AKI [5,11–13]. For survivors of out-of-hospital cardiac arrest, the area under the curve (AUC) for AKI development was reported at 0.97[10]. Furthermore, urinary levels of [TIMP-2]• [IGFBP7] exceeding 2.0 (ng/mL) have shown predictive significance for the need for kidney replacement therapy and short-term mortality [14–16]. Most research has concentrated on the predictive value of [TIMP-2]• [IGFBP7] in single-cause AKI, with limited studies assessing whether its predictive efficacy varies across different AKI etiologies [4]. AKI, leaving the potential differences between AKI of varying etiologies largely unexplored.

This study aims to investigate potential variations in the predictive value of urinary [TIMP-2]• [IGFBP7] among patients from distinct cohorts (stroke, sepsis, and post-cardiac surgery) and to develop a comprehensive predictive model that incorporates risk factors alongside urinary [TIMP-2]• [IGFBP7] for AKI. The study will also evaluate the predictive value of urinary [TIMP-2]• [IGFBP7] for continuous renal replacement therapy (CRRT) and 28-day mortality within each cohort.

## 1 Methods

### 1.1 Study design and participants

**Fig 1:**
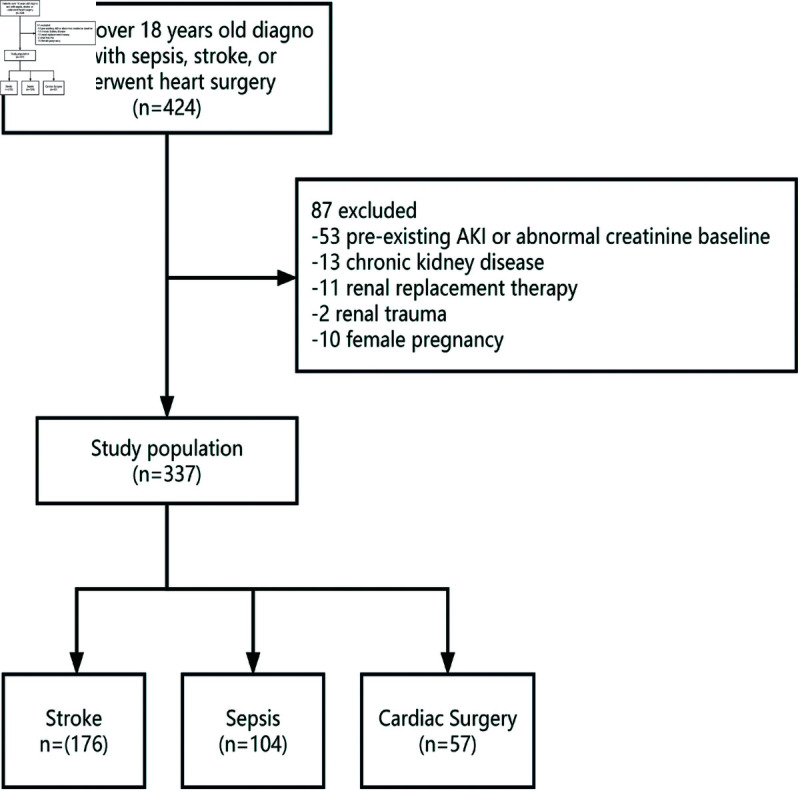
Flow chart of patients. AKI indicates acute kidney injury.

This single-center prospective observational study was designed to evaluate the predictive capacity of urinary [TIMP-2]• [IGFBP7] across three distinct etiologies of AKI. From November 1, 2019, to October 30, 2021, 337 adult patients in the ICU were recruited from Zhong Nan Hospital at Wuhan University in China (Fig 1).

Eligible participants were adults (aged over 18 years) who were admitted to the ICU with diagnoses of stroke, sepsis, or post-cardiac surgery, and whose onset or surgical intervention occurred within 24 hours. The study was approved by the Bioethics Committee of the Ethics Committee at Zhongnan Hospital of Wuhan University (No. 2017004). Written informed consent was obtained from all participants or their legally authorized representatives. Exclusion criteria encompassed abnormal baseline serum creatinine levels (>1.5 mg/dL or eGFR <60 mL/min/1.73 m^2^) at ICU admission, previous AKI diagnoses, chronic kidney disease (CKD), renal replacement therapy before ICU admission, renal trauma, and pregnancy. Urine samples for assessing urinary [TIMP-2]⋅[IGFBP7] were collected four hours after ICU admission and stored at freezing temperatures. The primary outcome measured was the incidence of AKI. Short-term prognostic indicators included the occurrence of AKI within seven days, the utilization of CRRT over seven days, and mortality rates at 28 days.

### 1.2 Samples and data collection

Urine specimens were obtained using a sterile catheter, centrifuged at 1200 rpm for 10 minutes, and the supernatant was aspirated for storage at -80 ^°^C and thawed immediately before analysis. Relevant clinical information, including medical history, demographics, laboratory results, treatment details, and participant characteristics within each subgroup, was extracted from hospital records. TIMP-2 and IGFBP7 levels were quantified using ELISA kits sourced from Wuhan Gemei Biotechnology. The concentrations of TIMP-2 and IGFBP7 were multiplied and divided by 1000 to yield [TIMP-2]• [IGFBP7], reported as (ng/ml)^2^/1000.

### 1.3 Measurement of kidney function and study definition

Serum creatinine levels, urine output, and follow-up data were retrieved from electronic medical records. The estimated glomerular filtration rate (eGFR) was calculated using the Chronic Kidney Disease Epidemiology Collaboration equation. Acute kidney injury was defined per the Kidney Disease: Improving Global Outcomes (KDIGO) Work Group criteria as an absolute increase in creatinine of at least 0.3 mg/dL (30 *μ*mol/L) within the first 48 hours post-ICU admission or procedure, a relative increase of at least 50% in creatinine within the first 7 days, or the initiation of dialysis. Baseline creatinine measurements were taken immediately before the procedure or upon ICU admission. Stroke diagnosis was based on patient symptoms, clinical signs, and brain imaging (CT or MRI) indicating ischemic or hemorrhagic stroke. Sepsis was defined according to Sepsis 3.0 criteria, with a SOFA score exceeding 2 for patients with suspected infection. Cardiac surgery was characterized by the necessity for cardiopulmonary bypass (CPB), encompassing valve replacement surgery, coronary artery bypass grafting (CABG), and combined CABG and valve replacement procedures.

### 1.4 Statistical analysis

The Shapiro-Wilk test assessed the normality of all numerical variables. Normally distributed data were expressed as mean ± standard error (X±S) and compared using the independent sample t-test. Non-normally distributed data were presented as the median and interquartile range (IQR) and analyzed via the Mann-Whitney U test. Categorical data were reported as frequency and percentage, and differences across groups were analyzed using the chi-square test. We adjusted for covariates and used logistic regression to assess the association between urinary [TIMP-2]• [IGFBP7] and the primary outcome of acute kidney injury, as well as secondary outcomes of CRRT usage and 28-day mortality. The predictive value of urinary [TIMP-2]• [IGFBP7] was analyzed using receiver operating characteristic (ROC) curves, and the area under the ROC curve (AUC) was calculated. Validated risk factors for each outcome in each group were analyzed and transformed as appropriate, followed by multivariable logistic regression. Odds ratios with 95% confidence intervals (CIs) were reported. The AUCs for different types of AKI were compared using the DeLong test. Statistical analyses were performed using SPSS (version 23.0) and GraphPad (version 9.0). All tests were two-sided, and P < 0.05 was considered statistically significant.

## 2 Results

### 2.1 Patient characteristics

The research was carried out from November 1, 2019, to October 30, 2021, in the Intensive Care Unit at Zhong Nan Hospital of Wuhan University, China. A total of 424 patients were assessed, with 337 patients ultimately enrolled: 176 with stroke, 104 with sepsis, and 57 following cardiac surgery. Baseline characteristics were evaluated for both patients who developed Acute Kidney Injury (AKI) and those who did not (Table 1).

**Table 1:** Demographic and clinical characteristics of patients, stratified according to incidence of acute kidney injury.

Variables	Overall	AKI	No-AKI	P-value
	N=337	N=109	N=228	
Age—yr#	61±11.3	61.9±10.2	60.6±11.8	0.33
Male—No. (%)	177(52.5)	57(52.3)	120(52.6)	0.95
Hypertension—No. (%)	190(56.4)	65(59.6)	125(54.8)	0.41
Diabetes—No. (%)	103(30.6)	50(45.9)	53(23.2)	<0.01
CHD—No. (%)	66(19.6)	23(21.1)	43(18.9)	0.628
Stroke—No. (%)	176(52.2)	39(35.8)	137(60.1)	-
Sepsis—No. (%)	104(30.9)	52(47.7)	52(22.8)	-
Cardiac surgery—No. (%)	57(16.9)	18(16.5)	39(17.1)	-
Scr, *μ*mol/L^*^	61.4(45-77)	66(49-77.9)	59.35(43.2-76.5)	0.04
BUN, mmol/L#	5.4±1.59	5.4±1.56	5.3±1.64	0.84
HB, g/L #	117.7±23.7	114.4±21.7	119.3±24.5	0.08
APACHE II score^*^	18(15-21)	20(16-25)	18(14-21)	<0.01
28-day mortality—No. (%)	86(25.5)	45(41.3)	41(18.0)	<0.01
CRRT—No. (%)	29(8.6)	29(26.6)	0(0)	<0.01
[TIMP-2]•[IGFBP7], (ng/ml)2/1000^*^	0.47(0.25-0.83)	0.80(0.59-1.22)	0.33(0.21-0.60)	<0.01

^*^indicates that the SW test does not follow the normal distribution. # indicates that the SW test follows the normal distribution. Categorical variables are displayed as numbers and ratios of each cohort. Age, BUN, and Hb are means ± SD. Scr and APACHE II score are presented as median and interquartile range (IQR, 25th to 75th percentiles). Diabetes indicates diabetes mellitus, CHD indicates coronary heart disease, Scr indicates serum creatinine, BUN indicates blood urea nitrogen, Hb indicates hemoglobin, APACHEA II score indicates Acute Physiology and Chronic Health Evaluation II, CRRT indicates Continuous Renal Replacement Therapy, TIMP-2 indicates Human tissue inhibitor of metalloproteinases-2, IGFBP-7 indicates insulin-like growth factor binding protein 7.

The study included 337 patients with a mean age of 61 ± 11.3 years and a mean eGFR was 91.8 ± 17.6 ml/min/1.73m^2^. Among the patients who developed AKI (n=109), the mean age was 61.9 ± 10.2 years, and the mean eGFR was 87.6 ± 15.8 ml/min/1.73m^2^. In contrast, patients who did not develop AKI (n=228) had a mean age of 60.6 ± 11.8 years and a mean eGFR of 94.1 ± 18.9 ml/min/1.73m^2^. No statistically significant difference in age was observed between groups (P=0.33), but the AKI group demonstrated marginally lower baseline eGFR levels compared to the non-AKI group (P=0.04). Within this study, 86 patients succumbed, and 29 required CRRT. The AKI group included 39 individuals with stroke, 52 with sepsis, and 18 who underwent cardiac surgery. Of 109 AKI cases, 57 (52.3%) were stage 1, 23 (21.1%) stage 2, and 29 (26.6%) stage 3. Patients diagnosed with AKI exhibited a higher prevalence of diabetes mellitus and elevated APACHE II scores. No significant differences were noted in other baseline characteristics (p>0.05). The secondary outcome of 28-day mortality was significantly greater in the AKI cohort (45, 41.3%) compared to the non-AKI group (41, 18%).

### 2.2 Stroke, sepsis, and cardiac surgery cohorts

The demographic data and clinical characteristics of the cohorts with stroke, sepsis, and post-cardiac surgery are detailed in S1-S3 Tables. Statistically significant variables identified in the univariate analysis were incorporated as covariates in the binary logistic regression model ( Table 2).

**Table 2:** Multivariate analysis of risk factors for acute kidney injury.

Groups	Covariates	Unadjusted OR(95% CI)	P value^*^	Adjusted OR(95% CI)	P value^**^
ALL	[TIMP-2]• [IGFBP7]	11.16(5.81-21.43)	<0.01	16.82(7.99-35.44)	<0.01
	Diabetes	2.8(1.72-4.55)	<0.01	3.09(1.69-5.65)	<0.01
	APACHE II score	1.13(1.08-1.18)	<0.01	1.17(1.10-1.23)	<0.01
Stroke	[TIMP-2]• [IGFBP7]	10.15(4.47-23.06)	<0.01	13.07(4.12-41.44)	<0.01
	NIHSS score	1.20 (1.11-1.30)	<0.01	1.22 (1.09-1.37)	<0.01
	APACHE II score	1.09 (1.03-1.16)	<0.01	1.16 (1.05-1.28)	<0.01
	Sex	2.40 (1.08-5.30)	0.03	-	0.35
	Diabetes	3.83 (1.76-8.32)	<0.01	-	0.32
	Drinking	2.25 (1.07-4.72)	0.03	-	0.06
	Loop diuretics	3.45 (1.64-7.26)	<0.01	-	0.82
	vasoactive agent	4.04 (1.91-8.54)	<0.01	-	0.15
	Interventional opertion	0.37 (0.15-0.95)	0.04	-	0.13
	Tracheotomy	3.53 (1.53-8.15)	<0.01	-	0.12
Sepsis	[TIMP-2]• [IGFBP7]	40.89 (8.58-194.88)	<0.01	23.51(1.52-363.88)	0.02
	SOFA score	1.40 (1.21-1.63)	<0.01	1.39 (1.10-1.75)	<0.01
	PCT	1.08 (1.04-1.12)	<0.01	1.08 (1.03-1.14)	<0.01
	Hypertention	0.28 (0.12-0.67)	<0.01	-	-
	Diabetes	0.44 (0.20-0.98)	0.04	-	-
	APACHE II score	1.12 (1.09-1.30)	<0.01	-	-
	LAC	1.28 (1.02-1.60)	0.03	-	-
Cardiac surgery	[TIMP-2]• [IGFBP7]	35.20(4.43-279.98)	<0.01	206.71 (7.08-603.14)	<0.01
	APACHE II score	1.26 (1.10-1.45)	<0.01	1.51 (1.14-2.01)	<0.01
	EF	0.89 (0.82-0.98)	0.02	-	-
	EC	1.02 (1.00-1.04)	0.03	-	-
	MAP	0.84 (0.73-0.96)	0.01	-	-
	RBC transfusion	1.34 (1.06-1.70)	0.01	-	-

P values^*^ for comparing the OR with and without covariates; p values^**^ for comparing the Adjusted OR *with and without covariates. OR indicates odds ratio, CI indicates confidence interval, Diabetes indicates diabetes melitus, APACHE II score indicates Acute Physiology and Chronic Health Evaluation, NIHSS score indicates National Institute of Health stroke scale, SOFA score indicates Sequential Organ Failure Assessment score, PCT indicates procalcitonin, LAC indicates Lactic acid, EF indicates Ejection Fraction, EC indicates* extracorporeal circulation, MAP indicates mean arterial pressure, RBC transfusion indicates red blood cell transfusion.

After adjusting for these risk factors, APACHE II scores, urinary [TIMP-2]• [IGFBP7], and diabetes were found to elevate the risk of AKI across all people. In the stroke cohort, factors associated with AKI included the NIHSS score, APACHE II score, and urinary [TIMP-2]• [IGFBP7] (OR=13.07, 95% CI 4.12-41.43, P<0.01). In contrast, among the 104 sepsis patients admitted to the ICU, the identified risk factors were the SOFA score, PCT, and urinary [TIMP-2]• [IGFBP7] (OR=23.51, 95% CI 1.52-363.88, P<0.01). For the post-cardiac surgery cohort, the APACHE II score and urinary [TIMP-2]• [IGFBP7] (OR=206.71, 95% CI 7.08-603.14, P<0.01) were associated with an increased risk of AKI.

### 2.3 Performance of urinary [TIMP2]• [IGFBP7] and other indicators predicting AKI in different group

**Fig 2:**
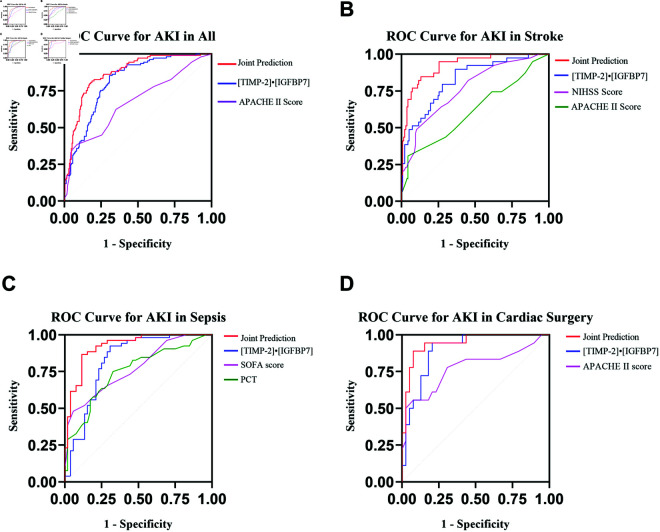
ROC curve for [TIMP2]• [IGFBP7] and joint prediction model predict the incidence of AKI in all people and three cohorts (stroke, sepsis and cardiac surgery) differing in AKI etiology. (**A**) ROCs showing the ability of [TIMP2]• [IGFBP7], APACHE II score , and [TIMP2]• [IGFBP7] + APACHE II score to predict AKI in all people. **(B)** ROCs showing the ability of [TIMP2]• [IGFBP7], NIHSS score, APACHE II score, and [TIMP2]• [IGFBP7] + NIHSS score + APACHE II score to predict AKI in the stroke group. **(C)** ROCs showing the ability of [TIMP2]• [IGFBP7], SOFA score, PCT, and [TIMP2]• [IGFBP7] + SOFA score + PCT to predict AKI in the sepsis group. **(D)** ROCs showing the ability of [TIMP2]• [IGFBP7], APACHE II score, and [TIMP2]• [IGFBP7] + APACHE II score to predict AKI in the cardiac surgery group.

The predictive performance of urinary [TIMP-2]• [IGFBP7] and other indicators for AKI across different subgroups and in combined prediction models is illustrated in (Table 3) and (Figure 2). In the post-cardiac surgery cohort, the area under the curve (AUC) for urinary [TIMP-2]• [IGFBP7] was 0.90 (95% CI 0.82-0.98). Despite this high AUC, Delong’s test revealed no significant difference in predictive capability between the cardiac surgery cohort and the stroke (P=0.20) or sepsis (P=0.21) cohorts. Furthermore, no significant difference was observed between the stroke and sepsis cohorts (P=0.95).

**Table 3:** Calculated AUC, sensitivities, specificities, predictive values, and compare the diagnostic efficiency of different models for AKI.

	Covariates	AUC(95%CI)	P value^*^	Best cut off	Sensitivity	Specificity	P value^**^
ALL	[TIMP-2]• [IGFBP7]	0.81(0.77-0.86)	<0.01	0.5	0.85	0.69	1
	APACHE II score	0.67(0.61-0.74)	<0.01	21.5	0.39	0.91	
	Joint prediction	0.87(0.83-0.91)	<0.01	0.31	0.82	0.81	
Stroke	[TIMP-2]• [IGFBP7]	0.83 (0.75-0.90)	<0.01	0.5	0.90	0.64	0.78
	NIHSS score	0.77(0.68-0.85)	<0.01	16	0.49	0.90	
	APACHE II score	0.62 (0.51-0.72)	0.03	23	0.31	0.96	
	Joint prediction	0.92(0.88-0.97)	<0.01	0.26	0.85	0.87	
Sepsis	[TIMP-2]• [IGFBP7]	0.82 (0.74-0.91)	<0.01	0.4	0.92	0.70	0.86
	SOFA score	0.77 (0.68-0.86)	<0.01	12.5	0.48	0.84	
	PCT	0.75 (0.65-0.84)	<0.01	15.1	0.77	0.67	
	Joint prediction	0.92(0.87-0.97)	<0.01	0.5	0.87	0.89	
Cardiac surgery	[TIMP-2]• [IGFBP7]	0.90 (0.82-0.98)	<0.01	0.36	0.94	0.70	0.08
	APACHE II score	0.78 (0.63-0.92)	<0.01	23.5	0.56	0.92	
	Joint prediction	0.94 (0.88-1.00)	<0.01	0.51	0.89	0.92	

p values^*^ for comparing the AUC with and without covariates; p values^**^ for comparing the AUC of [TIMP-2]• [IGFBP7] among three cohorts in AKI etiology. Joint prediction in each subgroup indicates [TIMP-2]• [IGFBP7] combined with the risk factors of each subgroup, which were identified by univariate analysis (Table 3) to predict the occurrence of AKI. AUC indicates area under the curve in receiver operating characteristics analysis, CI indicates confidence interval, NIHSS score indicates National Institute of Health stroke scale, SOFA score indicates Sequential Organ Failure Assessment score, and PCT indicates procalcitonin.

Regardless of the different AKI etiologies, combining urinary [TIMP2]• [IGFBP7] and APACHE II scores significantly improved ROC characteristics. ROCs show the ability of urinary [TIMP2]• [IGFBP7], APACHE II scores, and the joint prediction model to predict AKI for all people (Fig 2A). The combined prediction model 0.87(95% CI 0.83-0.91) demonstrated greater predictive values than individual predictions. The optimal cut-off value of urinary [TIMP-2]• [IGFBP7] was 0.5(ng/ml)^2^/1000, with a sensitivity of 0.85 and a specificity of 0.69.

In the stroke group, the ROC analysis (Fig 2B) shows the ability of urinary [TIMP-2]• [IGFBP7], NIHSS score, and APACHE II score to predict AKI. The combined prediction model, including urinary [TIMP-2]• [IGFBP7], NIHSS score, and APACHE II score, had an AUC of 0.92 (95% CI 0.88-0.97).

In the sepsis cohort, ROC analysis (Fig 2C) shows the predictive ability of urinary [TIMP-2]• [IGFBP7], SOFA score, and PCT. Combining urinary [TIMP-2]• [IGFBP7], SOFA scores, and PCT improved the predictive value for AKI, with an AUC of 0.92 (95% CI 0.87-0.97).

For patients who underwent cardiac surgery, ROC analysis (Fig 2D) shows the predictive ability of urinary [TIMP-2]• [IGFBP7] and APACHE II scores. The combined model of urinary [TIMP-2]• [IGFBP7] and APACHE II scores had an AUC of 0.94 (95% CI 0.88-1.00), significantly enhancing the predictive value for AKI.

### 2.4 Prognostic accuracy of urinary [TIMP-2]• [IGFBP7] in predicting AKI degree, CRRT and 28-days mortality

The urinary concentration of [TIMP-2]• [IGFBP7] demonstrated a positive correlation with the severity of AKI. In the stroke cohort, this biomarker exhibited a significant predictive capability for AKI grade, achieving an AUC of 0.94 (95% CI 0.89-0.98).

**Fig 3:**
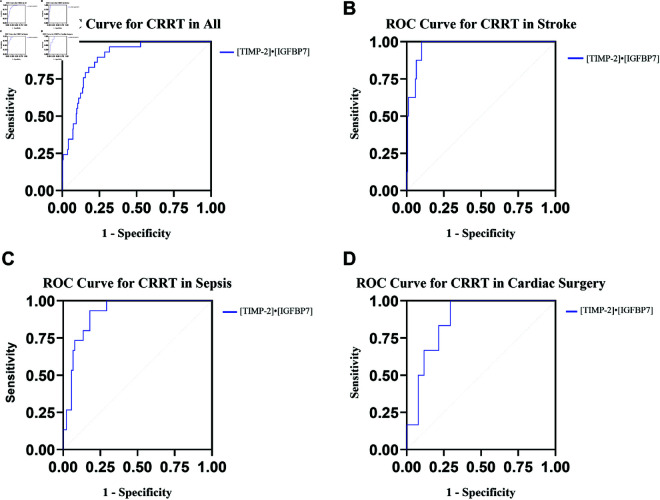
ROC curve for [TIMP2]• [IGFBP7] predicts the CRRT usage in all people and three cohorts (stroke, sepsis, and cardiac surgery) differing in AKI etiology. **(A)** ROC showing the ability of [TIMP2]• [IGFBP7] to predict CRRT usage in all people. **(B)** ROC showing the ability of [TIMP2]• [IGFBP7] to predict CRRT usage in the stroke group. **(C)** ROC showing the ability of [TIMP2]• [IGFBP7] to predict CRRT usage in the sepsis group. **(D)** ROC showing the ability of [TIMP2]• [IGFBP7] to predict CRRT usage in the cardiac surgery group. CRRT indicates continuous renal replacement therapy.

Among the 29 critically ill patients undergoing CRRT, the predictive value of urinary [TIMP-2]• [IGFBP7] for CRRT was determined to be 0.89 (95% CI 0.84-0.93) (Table 4). ROC analyses illustrated the predictive capacity of urinary [TIMP-2]• [IGFBP7] for CRRT across all patients, as well as within the stroke, sepsis, and cardiac surgery subgroups (Fig 3).

**Table 4:** Calculated AUC, sensitivities, specificities, predictive values of [TIMP-2]• [IGFBP7] for secondary outcomes AKI degree, CRRT usage, and 28-day mortality.

	Groups	AUC	P value	Best cut off	Sensitivity	Specificity
AKI degree	All	0.90(0.86-0.94)	<0.01	0.74	0.93	0.80
	Stroke	0.94 (0.89-0.98)	<0.01	0.981	0.93	0.83
	Sepsis	0.92(0.86-0.98)	<0.01	0.73	0.95	0.88
	Cardiac surgery	0.91(0.83-0.99)	<0.01	0.60	0.90	0.83
CRRT	All	0.89(0.84-0.93)	<0.01	0.74	0.90	0.76
	Stroke	0.97(0.94-1.00)	<0.01	1.23	1.00	0.90
	Sepsis	0.92 (0.86-0.97)	<0.01	0.73	0.93	0.82
	Cardiac surgery	0.87(0.77-0.98)	<0.01	0.39	1.00	0.71
28-day mortality	All	0.74(0.69-0.80)	<0.01	0.71	0.61	0.71
	Stroke	0.73 (0.65-0.81)	<0.01	0.337	0.89	0.47
	Sepsis	0.76(0.64-0.89)	<0.01	0.71	0.79	0.80
	Cardiac surgery	0.82(0.64-1.00)	<0.01	0.74	0.80	0.83

**Fig 4:**
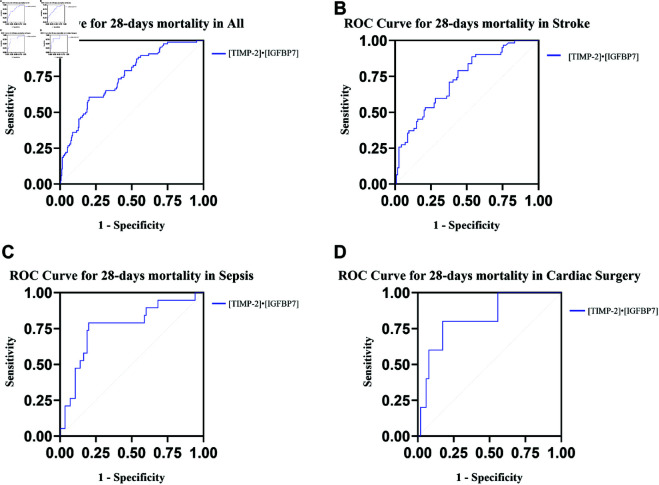
ROC curve for [TIMP2]• [IGFBP7] predicts the 28-day mortality in all people and three cohorts (stroke, sepsis, and cardiac surgery) differing in AKI etiology. **(A)** ROC showing the ability of [TIMP2]• [IGFBP7] to predict 28-day mortality in all people. **(B)** ROC showing the ability of [TIMP2]• [IGFBP7] to predict 28-day mortality in the stroke group. **(C)** ROC showing the ability of [TIMP2]• [IGFBP7] to predict 28-day mortality in the sepsis group. **(D)** ROC showing the ability of [TIMP2]• [IGFBP7] to predict 28-day mortality in the cardiac surgery group.

A total of 86 patients succumbed within 28 days, yielding a mortality rate of 25.5%, comprising 45 patients from the AKI cohort and 41 patients from the non-AKI cohort. The ROC curve for urinary [TIMP2]• [IGFBP7] concerning 28-day mortality across various subgroups is presented in Figure 4, underscoring its efficacy in diverse patient populations (Fig 4A-D).

## Discussion

This is the first study to compare the predictive value of [TIMP-2]• [IGFBP7] for AKI differing in AKI etiology. We evaluated the predictive efficacy of [TIMP-2]• [IGFBP7] concerning the incidence, severity, and short-term outcomes of AKI in three distinct cohorts of critically ill patients: those with stroke, sepsis, and post-cardiac surgery. Analyzing clinical data and urinary [TIMP-2]• [IGFBP7] concentrations from 337 high-risk ICU patients, we established that [TIMP-2]• [IGFBP7] exhibits robust predictive value for AKI across all groups. Furthermore, the integration of [TIMP-2]• [IGFBP7] with a risk factor prediction model substantially enhanced the predictive accuracy for AKI in high-risk patients. Nevertheless, the predictive performance within the cardiac surgery cohort did not significantly surpass that of the stroke (P=0.20) and sepsis (P=0.21) cohorts. While DeLong’s test showed no statistical significance (P>0.20), the numerically higher AUC for cardiac surgery (0.90) versus sepsis (0.82) may reflect etiology-specific biomarker performance. The 0.08 difference could be clinically meaningful, as prior decision analyses demonstrate that modest AUC increases (e.g., 0.03-0.05) may impact risk stratification [17]. This trend warrants validation in larger cohorts. The overlapping 95% CIs (cardiac surgery: 0.82–0.98; sepsis: 0.74–0.91) suggest biomarker performance may be comparable across etiologies in real-world settings where precision is limited.

According to KDIGO criteria, the prevalence of AKI among stroke patients ranges from 20.3% to 33% [18,19], which exceeds that of the general hospitalized population and may be attributed to brain-kidney interactions[20]. Elevated APACHE II and NIHSS scores emerged as independent risk factors for AKI in this cohort. High APACHE II scores are generally associated with poor prognosis in patients with kidney injury and stroke [21–23]. Consistent with our findings, prior studies have identified an increased NIHSS score as a risk factor for AKI, potentially indicating the severity of dysphagia and fluid circulation complications [24,25]. In the stroke cohort, [TIMP-2]⋅[IGFBP7] demonstrated a high AUC value of 0.94 for predicting AKI severity, indicating its potential clinical utility in this context.

Inflammatory injury is a significant contributor to septic AKI [26,27]. Previous studies have shown that PCT and SOFA scores are independent risk factors for septic AKI [28,29], which aligns with our findings. SOFA scores reflect organ function status and disease severity, while PCT levels are related to the degree of infection. The inflammatory cascade response plays a crucial role in septic AKI.

Cardiac surgery represents the second most significant risk factor for AKI in ICU patients, with incidence rates varying widely from 5% to 42% due to variations in surgical procedures [30,31]. Independent risk factors for AKI after cardiac surgery included APACHE II scores. Additional factors such as preoperative diabetes, hypertension, reduced left ventricular ejection fraction, intraoperative cardiopulmonary bypass duration, mean arterial pressure, and volume of red blood cell transfusion contribute to the development of AKI [32,33]. However, these factors did not emerge as independent risk factors for AKI in our investigation, likely because APACHE II scores may partially reflect the patients’ overall condition. The limited sample size of cardiac surgery patients may have constrained the analysis of risk factors.

Prior research has established the optimal cut-off value for [TIMP-2]• [IGFBP7] at 0.3 (ng/ml)^2^/1000 [8,34,35]. However, the overall optimal truncation value of [TIMP-2]• [IGFBP7] for AKI in our study was slightly higher than in previous studies [36]. This discrepancy may be attributed to the heightened baseline levels of [TIMP-2]• [IGFBP7] observed in ICU patients, as well as the increased prevalence of severe AKI cases [35,37]. Differences in population demographics may also contribute to varying detection indexes. In China, the optimal cut-off value ranges from 0.41 to 0.98 (ng/ml)^2^/1000, which aligns with our findings. Consequently, larger-scale studies are warranted to further elucidate the impact of population heterogeneity on baseline concentrations of [TIMP-2]• [IGFBP7] in patients.

In the Tribes-AKI study, the integration of IL-8, NGAL, Renal Resistance Index (RRI), and cell cycle arrest biomarkers enhanced the prediction of AKI [38,39]. Our findings suggest that combining [TIMP-2]• [IGFBP7] with independent risk factors further optimizes AKI diagnosis. The human body functions as an integrated and cohesive system, with various organs interacting to different extents, such as the renal-brain and renal-lung interactions [40,41]; therefore, targeted joint prediction models can substantially enhance the predictive capability of AKI across different etiologies. Consequently, the development of a comprehensive prediction model that incorporates critical disease scoring systems alongside renal biomarkers holds considerable clinical significance.

CRRT can be effectively administered in patients experiencing hemodynamic instability, offering a significant benefit in the ICU [12]. While the reliability of biomarkers for predicting CRRT initiation may be debatable, our results indicate that [TIMP-2]• [IGFBP7] could serve as an early indicator for the commencement of CRRT. [TIMP-2]⋅[IGFBP7] showed a high AUC value of 0.89 for predicting the need for CRRT, which may help identify high-risk patients earlier. Moreover, [TIMP-2]⋅[IGFBP7] had a moderate AUC value of 0.74 for predicting 28-day mortality, suggesting its potential value in predicting short-term outcomes.

Our combined prediction model provides clinicians with a powerful tool to identify the risk of AKI in high-risk patients early, thereby enabling timely interventions to improve patient outcomes. Future research will focus on developing user-friendly tools and validating their effectiveness in diverse clinical settings.

## Limitations

Our study has several limitations. (1)Urine samples were collected only at recruitment without dynamic surveillance. Future studies should include sample detection at multiple time points to evaluate dynamic changes in [TIMP-2]• [IGFBP7]; (2)No follow-up was conducted regarding long-term outcomes, particularly the incidence of CKD; (3)This investigation was conducted at a single center with a limited sample size. Larger, multi-center cohort studies are necessary to enhance the findings; (4)Post-hoc power analysis revealed that the sample size, particularly in the cardiac surgery cohort, was limited in detecting significant differences with greater precision. Future studies should aim for larger cohorts to address these limitations and provide more robust validation of our findings.

## Conclusion

The predictive capacity of [TIMP-2]• [IGFBP7] for AKI in high-risk populations, including those with stroke, sepsis, and post-cardiac surgery, did not show significant variation. However, integrating urinary [TIMP-2]• [IGFBP7] levels with AKI risk factors markedly enhances the predictive accuracy for AKI.

## Supporting information

S1-S3 **Supplementary Table 1:** Characteristics of the stroke group. **Supplementary Table 2** : Characteristics of the sepsis group. **Supplementary Table 3** : Characteristics of the cardiac surgery group.
(PDF)
