# Supplementary material for: Predictive value of urinary [TIMP-2]•[IGFBP7] for AKI among sepsis, stroke, and cardiac surgery cohorts: A prospective study
Source: PLoS One. 2025 Oct 10;20(10):e0332272. doi: 10.1371/journal.pone.0332272 (PMC12513614; doi:10.1371/journal.pone.0332272)
Supplement: S1-S3 — Supplementary Table 1: Characteristics of the stroke group. Supplementary Table 2 : Characteristics of the sepsis group. Supplementary Table 3 : Characteristics of the cardiac surgery group. (PDF) [file pone.0332272.s001.pdf]

**Supplementary Table 1** Characteristics of the stroke group

| Variables | All (n=176) | AKI (n=39,22.2%) | No-AKI (n=137,77.8%) | P value |
|---|---|---|---|---|
| Demographic characteristic | | | | |
| Age# | 62 (11.6) | 64 (10.9) | 61 (11.8) | 0.24 |
| Male | 104 (59.1) | 29 (74.4) | 75 (54.7) | 0.03 |
| Stroke Type | | | | |
| IS | 113 (64.2) | 30 (76.9) | 83 (50.5) | 0.06 |
| HS | 63 (35.8) | 10 (25.6) | 53 (38.7) | 0.13 |
| Comorbidity | | | | |
| Hypertension | 139 (79.0) | 34 (87.2) | 105 (76.6) | 0.15 |
| Diabetes | 40 (22.7) | 17 (43.6) | 23 (16.8) | ＜0.01 |
| Coronary heart disease | 19 (10.8) | 4 (10.5) | 15 (11.0) | 0.90 |
| Atrial fibrillation | 8 (4.5) | 1 (2.56) | 7 (5.14) | 0.68 |
| Habits | | | | |
| Drinking | 52 (29.5) | 17 (43.6) | 35 (25.5) | 0.03 |
| Smoking | 49 (27.8) | 10 (25.6) | 39 (28.5) | 0.72 |
| Therapy | | | | |
| Iodine contrast agent | 90 (51.1) | 20 (51.3) | 70 (51.1) | 0.98 |
| Mannitol | 162 (92.0) | 127 (92.7) | 35 (89.7) | 0.55 |
| Loop diuretics | 52 (29.5) | 20 (51.3) | 32 (23.4) | ＜0.01 |
| Vancomycin | 4 (2.3) | 1 (2.5) | 3 (2.2) | 0.89 |
| Vasoactive drug | 67 (38.1) | 25 (64.1) | 42 (30.7) | ＜0.01 |
| Craniotomy | 78 (44.3) | 17 (43.6) | 61 (44.5) | 0.91 |
| Interventional operation | 51 (29.0) | 6 (15.4) | 45 (32.8) | 0.03 |
| Non-invasive mechanical ventilation | 69 (39.2) | 17 (43.6) | 52 (38.2) | 0.52 |
| Tracheal intubation | 59 (33.5) | 18 (46.1) | 41 (30.1) | 0.06 |
| Tracheotomy | 30 (17) | 13 (33.3) | 17 (12.4) | ＜0.01 |
| NIHSS score△ | 13 (11-16) | 16 (13-20) | 12 (9-16) | ＜0.01 |
| APACHE II score△ | 18 (15-21) | 19 (16-25) | 18 (15-21) | 0.03 |
| MAP△ | 118(109-132.5) | 119 (106.5-132.5) | 118(110-130.75) | 0.63 |
| Laboratory examination | | | | |
| White blood cell△ | 10.1 (8.1-13.1) | 11.4 (8.7-14.2) | 10.1 (7.9-13.0) | 0.37 |
| Hemoglobin g/L△ | 124.5 (21.8) | 125.7 (26.2) | 125.4 (28.0) | 0.52 |
| BUN△ | 5.3 (1.6) | 5.3 (2.0) | 5.4 (1.5) | 0.84 |
| Creatinine, μmol/L△ | 70.9 (16.0) | 71.27 (15.1) | 70.8 (16.3) | 0.87 |
| [TIMP-2]•[IGFBP7]△ | 0.48 (0.27-0.86) | 1.14 (0.63-1.69) | 0.38 (0.25-0.65) | ＜0.01 |
| CRRT | 8 (4.5) | 8 (20.5) | 0(0) | ＜0.01 |
| 28-day mortality | 62 (35.2) | 26 (66.7) | 36 (26.3) | ＜0.01 |

△indicates that the SW test does not follow the normal distribution, # indicates that the SW test follows the normal distribution

**Supplementary Table 2** Characteristics of the sepsis group

| Variable | All (n=104) | AKI (n=52,50%) | No-AKI (n=52，50%) | P value |
|---|---|---|---|---|
| Demographic characteristic | | | | |
| **Age**△ | 60 (55-71) | 61 (56-71) | 60 (54-72) | 0.72 |
| Male | 43 (41.3) | 20 (38.5) | 23 (44.2) | 0.55 |
| Comorbidity | | | | |
| Hypertension | 34 (32.7) | 24 (46.2) | 10 (19.2) | ＜0.01 |
| Coronary heart disease | 26 (35) | 16 (30.8) | 10 (19.2) | 0.17 |
| Diabetes | 40 (38.5) | 25 (48.1) | 15 (28.8) | 0.04 |
| Cerebrovascular disease | 33 (31.7) | 17 (32.7) | 16 (30.8) | 0.83 |
| Tumor | 40 (38.5) | 21 (40.4) | 19 (36.5) | 0.68 |
| Habit | | | | |
| Smoking | 31 (29.8) | 13 (25) | 8 (18.4) | 0.28 |
| Drinking | 34 (32.7) | 18 (34.6) | 16 (30.8) | 0.68 |
| SOFA score△ | 9 (7-13) | 12 (8-14) | 8 (6-10) | ＜0.01 |
| APACHE II score△ | 18 (14-23) | 20 (16-25) | 15 (13-18) | ＜0.01 |
| Laboratory examination | | | | |
| WBC△ | 13.8 (9.8-17.6) | 14.4 (10.8-18.1) | 12.6 (9.4-16.9) | 0.14 |
| NEC△ | 11.6 (8.3-15.3) | 11.9 (9.2-15.3) | 10.7 (8.0-14.8) | 0.20 |
| PLT# | 107.7 (48.1) | 100.3 (44.8) | 115 (50.5) | 0.12 |
| HGB# | 107.3(18.6) | 107.6 (18.4) | 107 (19) | 0.87 |
| ALT△ | 73.5 (38.3-106.5) | 76.5 (39.3-105) | 71(37.3-109.2) | 0.92 |
| AST△ | 62.5 (42-94.8) | 54.5 (36.8-92.3) | 69 (43-105.8) | 0.15 |
| Alb△ | 28.1 (24.5-33.9) | 27.8 (23.8-33) | 28.3 (24.6-34.3) | 0.87 |
| Scr# | 60.7 (20.7) | 61.3 (20.8) | 61.0 (20.7) | 0.77 |
| BUN# | 5.4 (1.6) | 5.3 (1.6) | 5.5 (1.6) | 0.57 |
| IL-6△ | 124.9 (68.1-617.6) | 122.3 (45.2-611.2) | 126.7 (78-633.4) | 0.46 |
| Lac△ | 2.5 (1.7-4.2) | 2.8 (1.7-4.5) | 2.1 (1.5-3.3) | 0.04 |
| PCT△ | 15.7 (8.9-28.6) | 24.1 (15.3-36.4) | 11.3 (6.3-18.9) | ＜0.01 |
| CRRT△ | 15 (14.4) | 15 (28.8) | 0 (0) | ＜0.01 |
| Length of ICU stay | 7 (4-9) | 8 (6-10) | 5 (3-7) | ＜0.01 |
| 28-day mortality | 19 (18.3) | 14 (26.9) | 5 (9.6) | 0.02 |
| [TIMP-2]•[IGFBP7]△ | 0.60 (0.20-0.83) | 0.71 (0.61-0.99) | 0.20 (0.14-0.58) | ＜0.01 |

△indicates that the SW test does not follow the normal distribution, # indicates that the SW test follows the normal distribution；

**Supplementary Table 3** Characteristics of the cardiac surgery group

| Variable | All (n=57) | AKI (n=18,31.6%) | No-AKI (n=39，68.4%) | P value |
|---|---|---|---|---|
| Demographic characteristic | | | | |
| Age# | 54.3 (10.9) | 54.9 (8.4) | 54.0 (11.9) | 0.76 |
| Male# | 30 (52.6) | 8 (44.4) | 22 (56.4) | 0.40 |
| Body mass index(BMI)# | 24 (3.7) | 24.3 (4.3) | 23.8 (3.4) | 0.66 |
| Comorbidity | | | | |
| Hypertension | 17 (29.8) | 7 (43.8) | 10 (25.6) | 0.31 |
| Diabetes | 23 (40.1) | 8 (50.0) | 15 (38.5) | 0.67 |
| Coronary heart disease | 21 (36.8) | 43.8 (7) | 14 (35.9) | 0.83 |
| Cerebrovascular disease | 17 (29.8) | 6 (37.5) | 11 (28.2) | 0.69 |
| Habit | | | | |
| Smoking | 17 (29.8) | 3 (18.8) | 14 (35.9) | 0.14 |
| Drinking | 17 (29.8) | 6 (37.5) | 11 (28.2) | 0.69 |
| EF△ | 59 (52-62) | 53 (48-61) | 60 (55-62) | 0.04 |
| Scr# | 64.3 (21.6) | 70.3 (21.8) | 61.6 (21.2) | 0.15 |
| BUN△ | 5.2(4.1-6.2) | 5.4(4.9-6.1) | 5.06(4.1-6.4) | 0.42 |
| WBC△ | 9.9(8.4-12.5) | 10.6(8.8-12.9) | 9.3(8.2-12.0) | 0.21 |
| HGB△ | 125.6(94.2-139) | 100(84.5-130.5) | 127.8(98.5-139) | 0.10 |
| Cardiopulmonary bypass time# | 88.4 (38.7) | 105.7 (35.8) | 80 (37.9) | 0.02 |
| Mean arterial pressure# | 62.3 (4.8) | 59.8 (4.4) | 63.4 (4.6) | ＜0.01 |
| Red blood cell infusion# | 6.0 (2.8) | 7.4 (3.1) | 5.3 (2.4) | ＜0.01 |
| Mechanical ventilation time# | 7.4 (2.7) | 7.0 (2.6) | 7.5 (2.7) | 0.49 |
| APACHE II score△ | 18 (14-23) | 25 (19-26) | 16 (14-19) | ＜0.01 |
| ICU stay time△ | 2 (1-3) | 3 (2-5) | 2 (1-2) | ＜0.01 |
| CRRT | 6 (10.5) | 6 (37.5) | 0 (0) | ＜0.01 |
| 28-day mortality | 5 (8.8) | 4 (25) | 1 (2.6) | 0.02 |
| [TIMP-2]•[IGFBP7]△ | 0.33 (0.21-0.69) | 0.78 (0.39-1.16) | 0.23 (0.19-0.35) | ＜0.01 |

△indicates that the SW test does not follow the normal distribution, # indicates that the SW test follows the normal distribution；
